# Localization and sub-cellular shuttling of HTLV-1 Tax with the RNAi machinery component Drosha

**DOI:** 10.1186/1742-4690-8-S1-A159

**Published:** 2011-06-06

**Authors:** Rachel Van Duyne, Irene Guendel, Mudit Tyagi, Aarthi Narayanan, Zachary Klase, Kylene Kehn-Hall, John Semmes, Fatah Kashanchi

**Affiliations:** 1 George Mason University, Department of Molecular and Microbiology, National Center for Biodefense and Infectious Diseases, Manassas, VA, 20110, USA; 2 The George Washington University Medical Center, Department of Microbiology, Immunology, and Tropical Medicine, Washington, DC, 20037, USA; 3 National Institutes of Health, Molecular Virology Section, Laboratory of Molecular Microbiology, NIAID, Bethesda, Maryland, 20892, USA; 4 Eastern Virginia Medical School, Department of Microbiology and Molecular Cell Biology, Cancer Biology and Infectious Disease Research Center, Norfolk, VA, 23508, USA

## 

The innate ability of the human cell to silence endogenous retroviruses through RNA sequences encoding microRNAs, suggests that the cellular RNAi machinery is a major mean by which the host mounts a response against contemporary retroviruses, such as HIV-1 and HTLV-1. Several recent publications have identified cellular miRNAs that target and hybridize to specific sequences of both the HIV-1 and HTLV-1 transcripts. However, much like the variety of host immune responses to retroviral infection, the virus itself contains mechanisms that assist in the evasion of viral inhibition through manipulation of the cellular RNAi pathway. Retroviruses can hijack both the enzymatic and catalytic components of the RNAi pathway, in some cases to produce novel viral miRNAs that can either assist in active viral infection or promote a latent state of infection. Here, we propose that HTLV-1 viral proteins contribute to the dysregulation of the RNAi pathway by altering expression of key components of the pathway. A survey of uninfected and infected cell lines revealed that Drosha was present at lower levels in all HTLV infected lines. Additionally, transfection of HeLa cells with Tax shows colocalization of Tax and Drosha in the nucleus (speckles), suggesting that the HTLV-1 viral transactivator physically interacts with Drosha and targets it to specific areas of the cell. This data suggests the direct interaction of HTLV-1 viral components with RNAi machinery proteins which may lead to their dysregulation in infected cells.

